# Attaining competency and proficiency in minimally invasive mitral valve repair: a learning curve assessment using cumulative sum analysis

**DOI:** 10.1186/s13019-023-02106-7

**Published:** 2023-01-05

**Authors:** Yue Shu, Yin Zheng, Shuwu He, Yiping Du, Dan Zhu, Zhensu Shi

**Affiliations:** 1 Department of Special Medical Services, Hainan ChengMei Hospital, Haikou, Hainan People’s Republic of China; 2 Department of Special Medical Services, Hainan Cancer Hospital, Haikou, Hainan People’s Republic of China; 3 Department of Cardiovascular Surgery, The Second Affiliated Hospital of Hainan Medical University, 48th of Bai Shui Tang Road, Haikou, 570311 Hainan People’s Republic of China; 4 Department of Cardiovascular Surgery, Fuwai Hospital, Beijing, People’s Republic of China

**Keywords:** Minimally invasive, Mitral valve repair, Learning curve, Cumulative sum analysis

## Abstract

**Objective:**

To evaluate the learning curve of minimally invasive mitral valvuloplasty (MVP).

**Background:**

Minimally invasive MVP is characterized by minimal trauma, minimal bleeding, and short postoperative recovery time. The learning curve of any new procedure needs to be evaluated for learning and replication. However, minimally invasive mitral valve technique is a wide-ranging concept, no further analysis of the outcomes and learning curve of minimally invasive Mitral valvuloplasty has been performed.

**Methods:**

One hundred and fifty consecutive patients who underwent minimally invasive MVP alone without concurrent surgery were evaluated. Using cardiopulmonary bypass (CPB) time and aortic clamping (AC) time as evaluation variables, we visualized the learning curve for minimally invasive MVP using cumulative sum analysis. We also analyzed important postoperative variables such as postoperative drainage, duration of mechanical ventilation, ICU stay and postoperative hospital stay.

**Results:**

The slope of the fitted curve was negative after 75 procedures, and the learning curve could be crossed after the completion of the 75th procedure when AC and CPB time were used as evaluation variables. And as the number of surgical cases increased, CPB, AC, postoperative drainage, duration of mechanical ventilation, ICU stay and postoperative hospital stay all showed different degrees of decrease. The incidence of postoperative adverse events is similar to conventional Mitral valvuloplasty.

**Conclusion:**

Compared to conventional MVP, minimally invasive MVP provides the same satisfactory surgical results and stabilization can be achieved gradually after completion of the 75th procedure.

## Introduction

Minimally invasive cardiac surgery has become a new trend in modern medicine because of its reduced trauma, less bleeding and shorter post-operative recovery time [1–3], and there has been a significant increase in the number of studies related to minimally invasive mitral valve surgery [4–7]. Since minimally invasive mitral valve surgery was first described by Cohn and Cosgrove in the mid-1990s, a variety of minimally invasive procedures, including the parasternal, hemi-sternotomy, mini-thoracotomy, totally robotic approach, and totally endoscopic approach, have been proposed [8]. Among them, Carpentier et al. successfully performed the first thoracoscopic-assisted Mitral valvuloplasty (MVP) in 1996 [9], and more and more surgeons are showing interest in thoracoscopic-assisted MVP. Using thoracoscopic techniques, the “direct view” of the valve can be clearly provided and the mitral valve and perivalvular structures can be better evaluated [10]. With the update of minimally invasive surgery technology, the totally thoracoscopic technique is more economical than the totally robotic approaches and less invasive than the thoracoscopic-assisted techniques [11]. However, to date, relatively few centers can carry out totally thoracoscopic MVP independently [12]. Increased postoperative complications, reduced repair quality and a longer learning curve are probably the main concerns regarding this technique. Minimally invasive mitral valve technique is a wide-ranging concept, yet there is no further subgroup analysis of its surgical outcomes and learning curve based on different procedures.

In this article, we summarize our clinical experience with 150 consecutives minimally invasive MVP and provide a comprehensive evaluation of the minimally invasive MVP technique using its CUSUM (cumulative summation) learning curve and early clinical outcomes, which in turn may help surgeons or centers in the early stages of learning the minimally invasive technique to improve the safety of the procedure and to benefit more patients.

## Materials and methods

### Study design

The research was conducted in accordance with the Declaration of Helsinki (revised 2013). The research was a retrospective clinical study without any specific interventions for the patients, all patients had previously approved the use of their medical records for research purposes, and the study protocol had been approved by local institutional review board. The informed consent form has been signed by the patients themselves or their immediate family members before the operation.

### Patients

From April 2016 to January 2022, a total of 150 minimally invasive MVPs were performed by an experienced surgeon in our medical centers. In order to reduce the effect of simultaneous surgery on cardiopulmonary bypass (CPB) time, aortic clamping (AC) time, patients with concurrent aortic valve surgery, congenital heart surgery, radiofrequency ablation of atrial fibrillation, mucosal aneurysm resection, combined severe coronary artery disease, and low ejection fraction (< 30%) were excluded. Specific details of the baseline information of the included patients are shown in Table 1.

**Table 1:** Preoperative baseline characteristics

Variable	Value
Male/female	93/57
Age	48.15 ± 15.875
Weight	65.71 ± 11.94
Height	165.61 ± 8.63
BMI	0.24 ± 0.034
*Comorbidities*	
CAD	16
AF	16
Hypertension	46
Diabetes	13
COPD	5
Chronic renal failure	18
EuroScore II	2.555 ± 2.44
*Echocardiographic data*	
LA, mm, mean ± SD	43.9 ± 4.84
LVDD, mm, mean ± SD	49.11 ± 5.241
LVEF, %, mean ± SD	64.91 ± 2.938
*NYHA CLASS grade*	
2	37
3	86
4	27
*Involved regions*	
Anterior leaflet (A1/A2/A3)	54 (13/24/17)
Posterior leaflet (P1/P2/P3)	74 (19/47/8)
Bivalvular leaflet	15
Commissure	7
Leaflet prolapse	112
*Etiology*	
Fibroelastic deficiency	101
Barlow’s disease	13
Congenital mitral cleft	12
Infective endocarditis	12
Rheumatic valve disease	10
Others	2

LA, Left atrial diameter; LVDD, Left ventricular dimension diastole; LVEF, Left ventricular ejection fraction

### Surgical technique

After the induction of general anesthesia, a left-sided double-lumen endotracheal tube was placed to allow for single-lung ventilation and defibrillator pads was placed across the chest. The patient was placed in a 30° left side position, a small pillow is placed under the scapula to open up the axillary space. A TEE probe was then placed to evaluate mitral valve and ventricular function in all patients before and after the procedure.

CPB was routinely established through the femoral artery, femoral vein, and right internal jugular vein. The the main port (approximately 2.5 cm) was located in the fourth intercostal space outside the right midclavicular line, through which the cardiac arrest fluid flushing tube was also passed. The thoracoscopy was placed through the third intercostal space on the right anterior axillary line, after inserting the aortic root cannula, while the left cardiac drainage and Chitwood clamp were punctured through the fourth intercostal space. At our center, MVP is performed using a conventional left atrial incision, parallel to the interatrial sulcus, and an artificial pneumothorax is routinely established using CO_2_.

### Data collection and analysis

Preoperative baseline information, intraoperative CPB time and AC time, postoperative bleeding, and incidence of surgical adverse events, including the presence of delayed extubation, secondary intubation, pleural effusion, pneumothorax, atrial fibrillation, poor incision healing, and stroke, were collected by the same physician.

Cumulative sum (CUSUM) is a statistical method that focuses on results rather than on the process of performing a program skill, it generates graphs that allow for quick detection of deviations from pre-established standards and is an alternative tool that can be used to evaluate the performance of individual programs [13]. CUSUM can be generated based on set acceptable and unacceptable failure rates and the degree to which type 1(α) and type 2(β) errors (false positive and false negative errors) will be tolerated [14]. CUSUM was defined as Sn = ∑(Xi − p0), where Xi = 0 for success and Xi = 1 for failure, p0 is the target reference [15]. In the CUSUM chart generated by Minitab Statistical Software (Version 20.3), surgical cases are arranged chronologically on the horizontal axis. The vertical axis f represents the cumulative total of AC time and CPB time, respectively. When the curve crosses the boundary, the complication rate of the surgeon is equal to or lower than the acceptable rate, which means that the learning curve is reached.

Perioperative data were analyzed using SPSS 26.0 software (SPSS Inc., Chicago, Illinois, USA). Continuous variables were expressed as means and standard deviations. Categorical variables were expressed as percentages. Continuous variables were compared using Student t-test and ANOVA analysis, and categorical variables were analyzed using chi-square test and Fischer’s exact test. And the significant differences were defined at *P* < 0.05.

## Results

### Learning curve

**Fig. 1:**
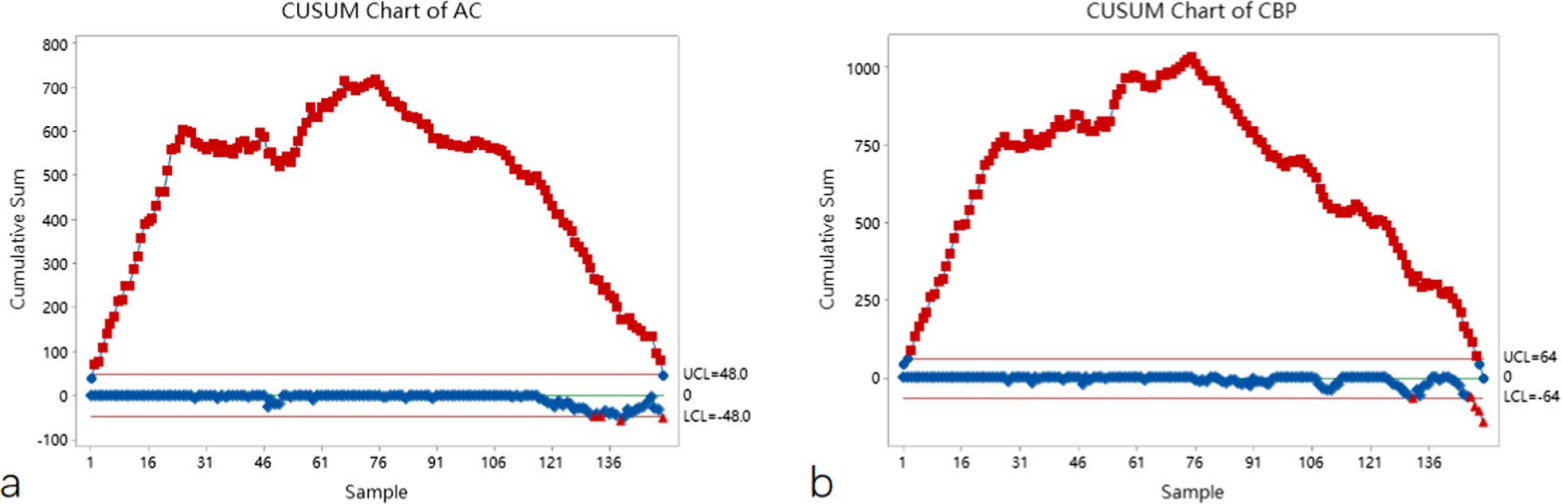
CUSUM curve analyses of the AC time (**a**) and CPB time (**b**)

All patients completed the surgery successfully, with no intermediate open-heart surgery and no perioperative deaths. Operation and postoperative characteristics are summarized in Table 2. The learning curve of AC and CPB visualized with CUSUM diagrams are shown in Fig. 1.

**Table 2:** Operation and postoperative characteristics

Variable	Group1	Group2	Group3	*P*
CPB (min)	168.82 ± 23.63	150.94 ± 18.83	138.86 ± 17.06	< 0.01
AC (min)	111.44 ± 19.943	101.92 ± 13.124	90.58 ± 10.433	< 0.01
*Surgical technique*				
Annuloplasty ring	49	49	50	> 0.99
Artificial chordae tendineae implantation	34	31	35	0.68
Commissuroplasty	5	7	8	0.67
Leaflet folding	3	5	4	0.93
Cleft suture	2	6	4	0.6
Edge to edge	0	1	0	–
Postoperative chest drainage in the first 24 h (ML)	244.4 ± 114.93	205.4 ± 95.238	129.8 ± 41.477	< 0.01
Mechanical ventilation length (H)	13.26 ± 8.179	11.05 ± 3.709	10.28 ± 3.297	0.022
ICU stay (D)	2.49 ± 1.968	2.08 ± 1.515	1.9 ± 1.099	0.164
Postoperative hospital stays (D)	6.64 ± 1.67527	5.36 ± 1.00529	5.14 ± 1.4287	> 0.01

**Fig. 2:**
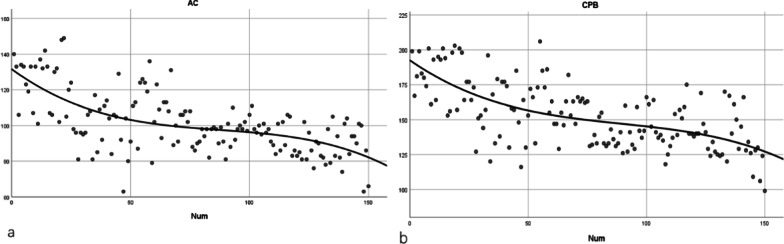
Time series chart of AC (**a**) and CPB (**b**) time

Each trend graph confirmed the presumed shape of an initially steep slope. The fitted equation for CPB is Y = 192.5462959411959 + − 1.173480390520274 * x + 0.01115565046303966 * x^2^ − 4.161350192966322e-005 * x^3^, R^2^ = 0.434, and the fitted equation for AC is Y = 131.5939091152518 + − 0.9761575056383154 * x + 0.01005722184889189 * x^2^ + − 3.832091085504812e-005 * x^3^, R^2^ = 0.412 (Fig. 2).

**Fig. 3:**
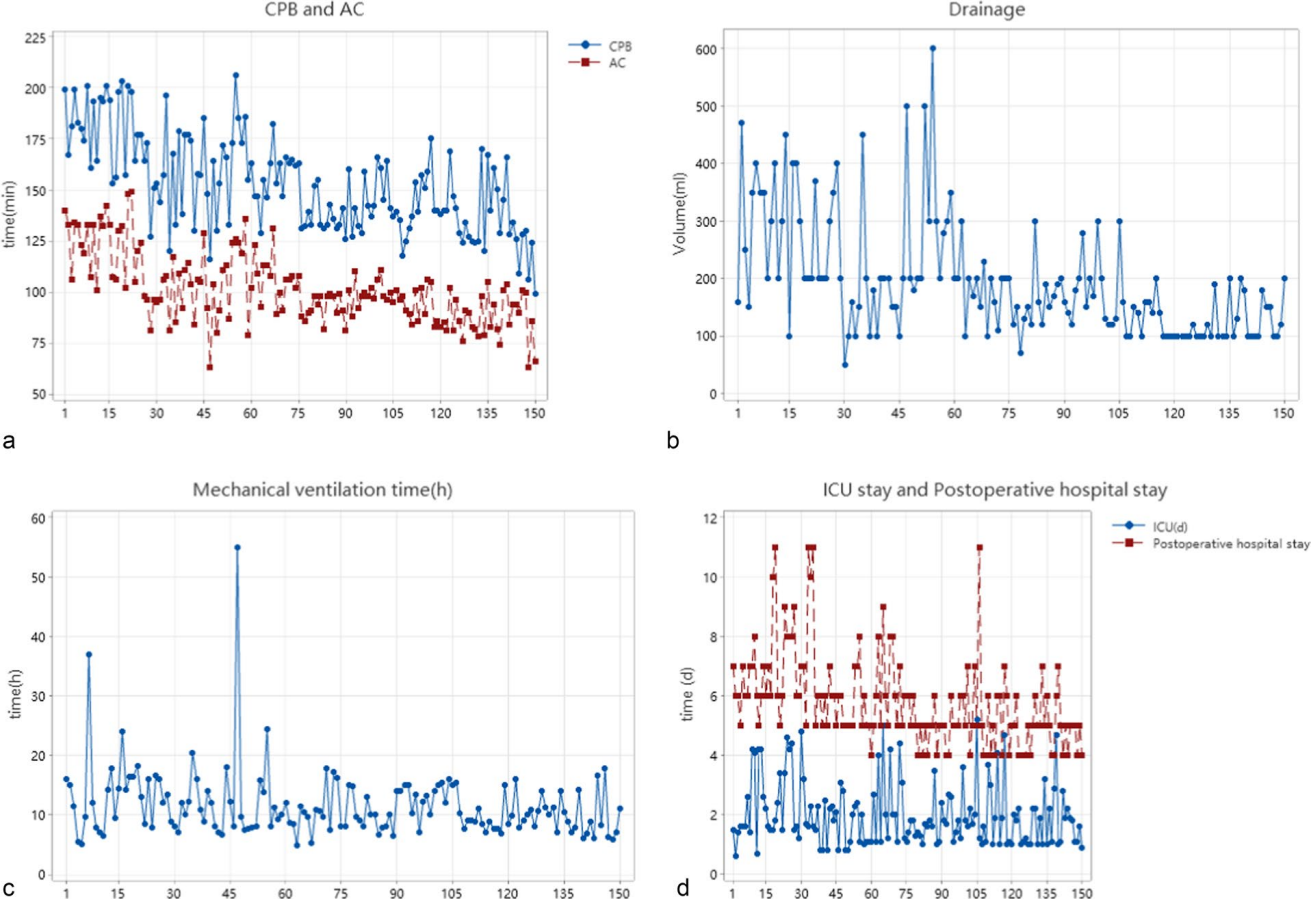
Time Series Chart of AC and CPB time (**a**), postoperative drainage (**b**), duration of mechanical ventilation (**c**), ICU stay and postoperative hospital stay (**d**)

As shown in the CUSUM plot, the slope of the fitted curve was negative after 75 procedures, so the learning curve could be crossed after completing 75 procedures. As the number of surgical cases increased, CPB, AC, postoperative drainage, duration of mechanical ventilation, ICU stay and postoperative hospital stay all showed different degrees of decrease, as shown in Fig. 3.

This statistical analysis showed a gradual decrease in the important variables toward a positive results trend as the accumulated number of cases increased in time. Time series plots were generated for visualization of the postoperative data, and we divided the enrolled patients into three groups (50/per group) chronologically for better intergroup comparison. Compared to the other variables that showed statistically significant differences, ICU stay, although not showing statistically significant differences, also showed a moderate decrease.

### Postoperative outcome

The overall postoperative adverse event incidence was 2.67%, as shown in the Table 3, none of the patients experienced serious complications including, for instance, postoperative death, cardiac rupture, pericardial tamponade and malignant arrhythmias, as well as respiratory failure, delayed extubation, secondary intubation, pleural effusion, pneumothorax, cerebral hemorrhage, pulmonary Infection, poor healing of incision and sternal dehiscence. IABP implantation was performed in two of the patients for transient decreased cardiac output, and the IABP was successfully removed. one patient underwent dialysis for renal insufficiency, and this patient had a preoperative history of renal insufficiency, and the intraoperative hypoperfusion may have exacerbated the renal impairment, one patient experienced stroke without disability.

**Table 3:** Early postoperative complications and Residual mitral regurgitation

Variable	Value
Serious complications	0
Secondary thoracotomy	0
Respiratory failure, n (%)	0
IABP implantation	2 (0.13%)
Delayed extubation	1 (0.6%)
Secondary intubation	0
Dialysis for renal failure	1 (0.6%)
Pleural effusion	0
Pneumothorax	0
Stroke	1(0.6%)
Cerebral hemorrhage	0
Pulmonary infection	5
Poor healing of incision	0
Sternal dehiscence	0
*Residual MR*	
0	94
1	33
2	18
3	4
4	1

## Discussion

Since the first successful thoracoscopic mitral valve surgery performed by Carpentier et al., minimally invasive valve surgery has made great progress in the last 25 years, including infrasternal small incision, direct right mini-incision and thoracoscopic-assisted right mini-incision, and robotic-assisted mitral valve surgery, all of which have been shown to have reduced trauma, decreased bleeding, and better recovery compared to traditional MV surgery [9]. With similar repair results, minimally invasive MVP could also be used for complex mitral valve lesions with the simultaneous advantages of minimally invasive valve surgery [16]. MVP is a more demanding technique compared with mitral valve replacement [17, 18], which is why in the early development of thoracoscopic Mitral valvuloplasty, high postoperative complications and prolonged operative time led some researchers to be suspicious of this technique [19].

When performing a new surgical procedure, surgeons need to understand the learning curve of the procedure in order to improve the learning efficiency of the procedure. By visualizing the learning curve, CUSUM realistically reflects the progression of a surgeon learning, practicing, and then plateauing for a new procedure, and eventually reaching stability. CUSUM could be used to provide continuous performance data and possibly to evaluate the training program themselves [14]. Since its first application in medical statistics by Bolsin S, CUSUM has been widely used in the evaluation of learning curves for various new procedures [13, 15]. By repeating and updating the learning curve after accumulating the variables one by one, the change pattern of the learning curve can be identified quickly. The CUSUM curve showed an upward slope when the operation time exceeded the mean value, and conversely, the CUSUM curve showed a downward slope. In this study, the CUSUM learning curve underwent a significant upward trend before the 75th surgery, and significant improvements in both AC and CPB were observed after the 75th surgery, which was similar to the results of previous learning curve studies of minimally invasive valve surgery, Although there were significant differences between different studies, the overall tendency of change in CUSUM was similar, with the number of procedures required to overcome the learning curve ranging from 64 to 116 [20–22]. However, learning curve outcomes may vary considerably between different minimally invasive valve procedures, with parasternal, hemi-sternotomy, and mini-thoracotomy approaches likely to reach plateau more rapidly, and correspondingly, totally robotic approaches, and totally endoscopic approach may require more time and training to achieve stability. However, previous studies related to learning curves of minimally invasive Mitral valvuloplasty have not performed more precise subgroup analyses according to the different procedures and did not make strict exclusions for simultaneous procedures, because any simultaneous procedure may result in prolonged AC and CPB times [20, 21, 23, 24]. In this study, the CUSUM of postoperative adverse events could not be visualized due to the low incidence, but according to previous literature, the incidence of postoperative complications was similar between minimally invasive MVP and conventional MVP surgery [25]. The CUSUM curves of AC and CPB showed a steep upward trend until the 26th surgery, followed by a slow upward and plateau period. This may be due to early re-familiarization and re-confirmation of the operative field and anatomy under fully thoracoscopic conditions as well as a slightly reduced level of cooperation, as fully thoracoscopic surgery may place higher demands on the assistants, anesthesiologists, and nursing team in addition to the surgeon, and each of these variables has its own learning curve.

Based on the valve surgery learning curve assessment, after completing a sufficient number of right heart valve procedures, one can attempt subsequently mitral valve surgery [26], which is also a process of re-familiarization with the cardiac anatomy. Therefore, we believe that the number of traditional MVPs previously completed by the surgeon also plays an important role in the morphology of the learning curve. In our study, the surgeon has independently performed more than 300 conventional MVP cases in the previous 5 years with favorable overall repair outcomes. It is worth mentioning that in our center, the categories of diseases the surgeons addressed were mainly coronary heart disease, followed by valvular disease and in some cases congenital heart disease. Therefore, the results of the study based on one non-valvular disease specialist may be some general applicability. In addition, maintaining a certain frequency of surgery may also have some effects on the morphology of the learning curve.

According to our clinical experience, an increasing number of patients requiring simultaneous surgery for tricuspid valve lesions, infective endocarditis, mucinous tumors, and atrial septal defects underwent minimally invasive MVP as the number of procedures increased, although we were unable to include patients with these concomitant procedures in the study given the homogeneity of the study population.

## Strengths and limitations

The advantage of this study over previous studies is that we set strict inclusion and exclusion criteria, and patients who underwent concurrent surgery such as atrial septal defect repair and tricuspid valve repair were strictly excluded, which to some extent reduced the effect of concurrent surgery on the target variables. Secondly, we exclusively evaluated the learning curve of minimally invasive mitral valvuloplasty, dividing the minimally invasive valve techniques further in detail, for the difference in learning curve between different minimally invasive approaches can be substantial.

This study also has some limitations. Firstly, this study is a retrospective study, and the selection of patients undergoing minimally invasive MVP may have undergone operator intervention, which may have influenced the subsequent CUSUM curves to some extent. Second, because of objective limitations, we were unable to propensity-match the study population to select patients who underwent conventional MVP with similar clinical baseline characteristics as a dyadic group for the control study. Maintaining a certain degree of frequency of minimally invasive surgeries may have a non-negligible impact on the learning curve, however, this may receive some limitations in terms of replication in other centers due to objective conditions.

## Data Availability

All data generated or analyzed during this study are included in this published article.
